# Supplementary material for: Maximal Respiratory Muscle Strength in Singaporean Adults: Normative Reference Values and Predictive Models from a Cross-Sectional Study
Source: Muscles. 2025 Oct 20;4(4):47. doi: 10.3390/muscles4040047 (PMC12550895; doi:10.3390/muscles4040047)
Supplement: Supplementary file 1 [file muscles-04-00047-s001.zip › muscles-3757228-supplementary.pdf]

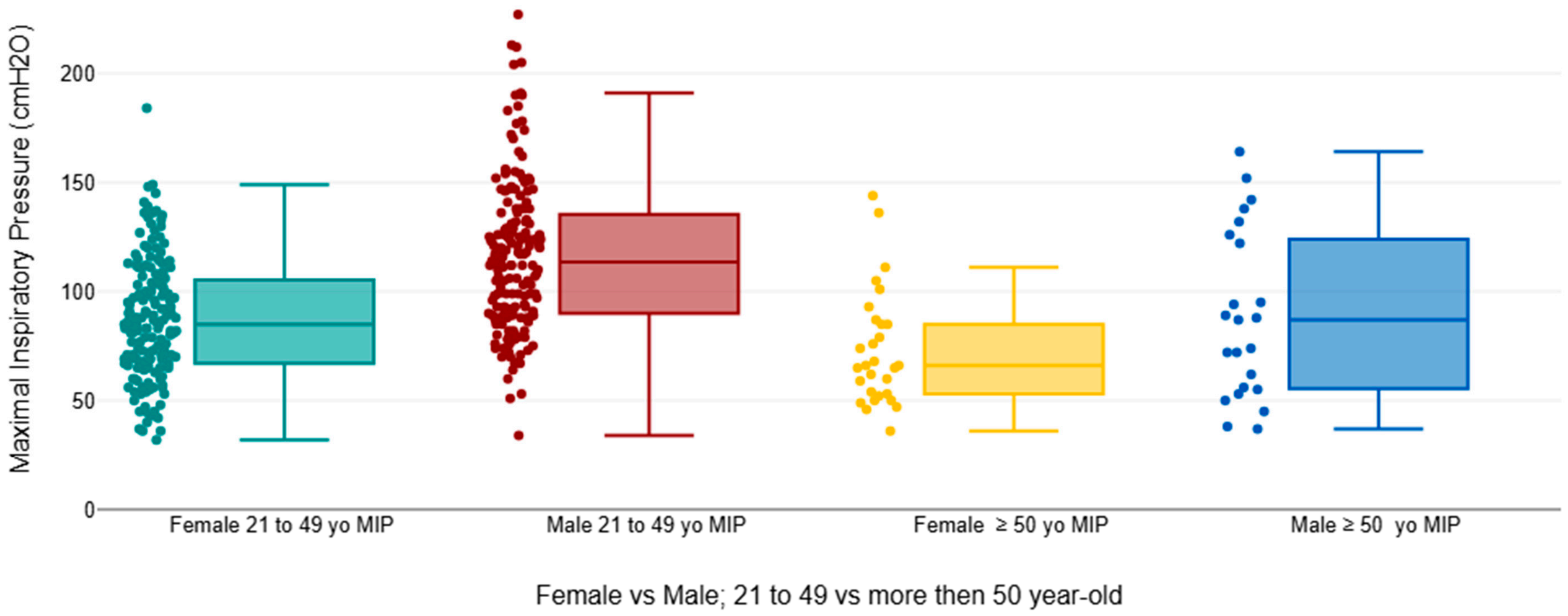


**Figure S1.** Maximal Inspiratory Pressure distribution across 21-49 years and over 50 years: Median and Maximum-Minium Range Comparison by Gender (Note: MIP: maximal inspiratory pressure; cmH2O: centimetre of water)

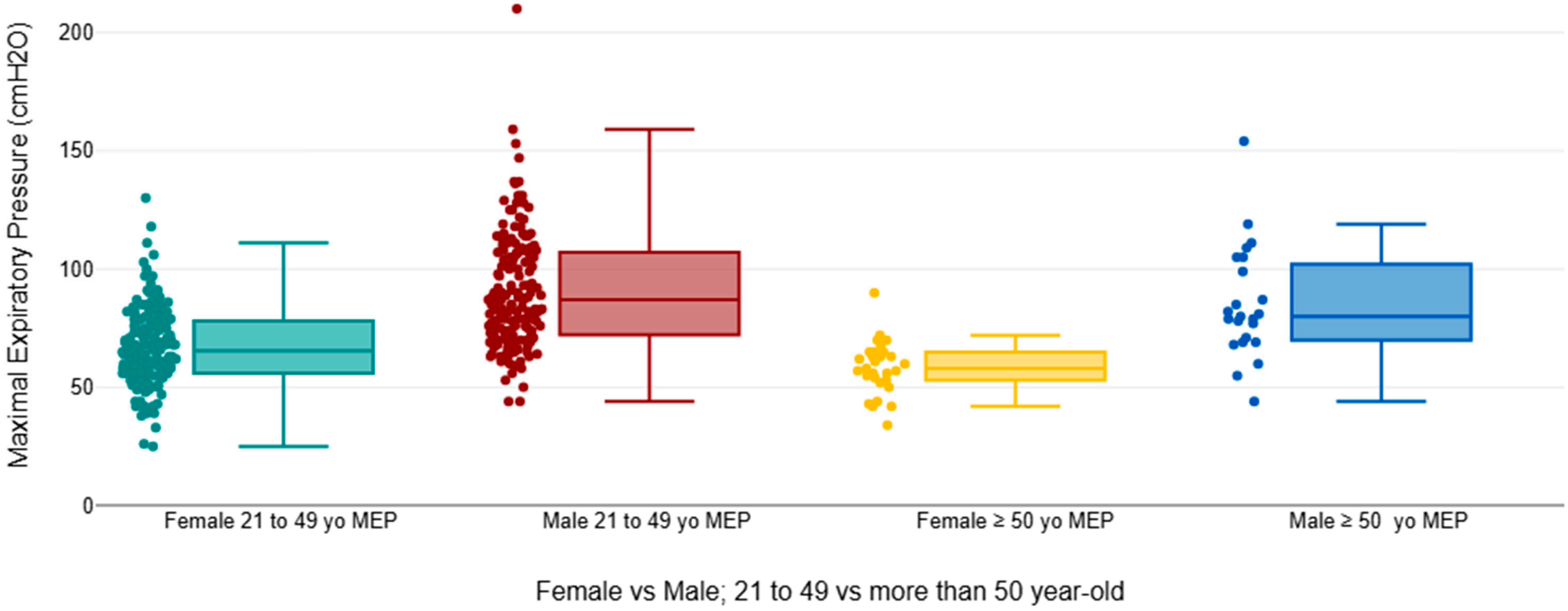


**Figure S2.** Maximal Expiratory Pressure distribution across 21-49 years and over 50 years: Median and Maximum-Minium Range Comparison by Gender (Note: MEP: maximal expiratory pressure; cmH2O: centimetre of water)
